# Performance of Crumb Rubber Tire-Modified Bitumen for Malaysian Climate Regions

**DOI:** 10.3390/ma17235800

**Published:** 2024-11-26

**Authors:** Ronald Blab, Juraidah Ahmad, Ekarizan Shaffie, Norbaya Sidek, Johannes Mirwald, Lukas Eberhardsteiner, Bernhard Hofko

**Affiliations:** 1 Institute of Transportation, Faculty of Civil and Environmental Engineering, TU Wien, Karlsplatz 13/E230, 1040 Vienna, Austria; 2 School of Civil Engineering, College of Engineering, Universiti Teknologi MARA, Shah Alam 40450, Selangor, Malaysiaeka@uitm.edu.my (E.S.); norbayasidek@uitm.edu.my (N.S.); 3 Institute for Infrastructure Engineering and Sustainable Management (IIESM), Universiti Teknologi MARA, Shah Alam 40450, Selangor, Malaysia

**Keywords:** bitumen modification, bitumen rheology, crumb rubber, design temperature, performance grade, tire recycling

## Abstract

Researchers are increasingly concerned about the vast amounts of waste rubber tires produced globally, which contribute significantly to environmental pollution. The potential of incorporating waste rubber tires to modify bitumen has garnered considerable interest. This study assesses pavement design temperatures according to SUPERPAVE standards for representative Malaysian regions. The assessment is based on hourly air temperature data and simulates temperature diffusion in typical Malaysian road pavements using the finite difference method (FDM). Tests on neat bitumen (PEN 60/70) and crumb rubber-modified bitumen (CR-TMB) samples evaluated their physical and rheological properties across various temperatures and aging stages. These tests were conducted using the dynamic shear rheometer, rotational viscometer, and bending beam rheometer. The attenuated total reflectance Fourier transform infrared spectroscopy (ATR-FTIR) analysis provided insights into the aging processes of both PEN 60/70 and CR-TMB. The findings indicate that adding 15% crumb rubber to produce CR-TMB enhances the physical and rheological properties of bitumen. Additionally, this modification significantly improves aging behavior, highlighting its potential for more resilient and sustainable road construction materials. Therefore, the use of crumb rubber in road construction should be considered to improve pavement durability and strength. Furthermore, utilizing crumb rubber as an alternative material can reduce costs by recycling waste materials.

## 1. Introduction

Depletion of natural resources and production of enormous quantities of waste materials are worldwide issues and must be addressed in sustainable development. The principles of sustainable development were introduced within the sustainable development goals (SDGs), which include decreasing environmental pollution and achieving sustainable and environmentally good management of all wastes. There has been a dramatic increase in the use of recycled materials/by-products as alternative eco-materials in the construction of pavement, e.g., [1].

Crumb rubber tire modified bitumen (CR-TMB) is a promising innovation in road construction, where waste tires are incorporated into bitumen to enhance pavement performance. Research on CR-TMB focuses on evaluating its mechanical properties, durability, and environmental impact [2,3,4,5,6,7,8,9]. This paper focuses on the assessment of the design temperatures for representative Malaysian asphalt pavements and the identification of the rheological properties of a CR-TMB formulation produced in Malaysia in order to investigate its long-term performance to promote sustainable and resilient infrastructure development.

Several publications investigated the rheological assessment of aged, rubberized bitumen. The findings revealed a noteworthy and significant impact of crumb rubber on bitumen rheology. This is because adding rubber can aid in lowering bitumen’s susceptibility to hardening and rate of hardening by substantially increasing the storage and loss moduli and decreasing the intensity of reduction in the storage modulus with temperature, e.g., [2,3,4]. In pavement engineering, CR-TMB is an area of growing interest for many researchers focusing on enhancing asphalt performance through the incorporation of recycled tire rubber [5,6,7,8].

These papers provide a comprehensive understanding of the various aspects of CR-TMB, from its mechanical properties to environmental benefits and long-term performance. The process of aging results in significant chemical and physical changes in bitumen, which subsequently impact the performance and overall durability of asphalt pavements. As summarized by Picado-Santos, et al. [9], the incorporation of crumb rubber into bitumen results in significant enhancements in its rheological properties.

The application of CR-TMB in tropical regions is recently receiving attention for its potential to enhance road durability under high temperatures and humid conditions. Tropical climates pose unique challenges for asphalt pavement due to intense heat, heavy rainfall, and rapid degradation and bitumen aging caused by UV exposure and oxidation. Studies indicate that CR-TMB improves the mechanical properties of hot mix asphalt (HMA), such as elasticity and toughness, which are crucial for handling heavy loads and high temperatures typical in tropical regions. For instance, the incorporation of crumb rubber enhances the pavement’s resistance to rutting and cracking, extending its lifespan in countries like Malaysia and Indonesia [10,11,12].

However, despite significant research on CR-TMB in a variety of climatic conditions, there is a noticeable gap in studies focused specifically on its application in tropical regions such as Malaysia. Therefore, understanding CR-TMB’s rheology and performance properties under these specific conditions is essential for determining its suitability in tropical regions, yet research on this topic remains limited.

## 2. Scope and Methodology

Within the scope of the present investigations, the performance properties of a typical neat bitumen (NB) used in tropical regions, which is modified in the course of the so-called wet process, are examined. In the wet process, crumb rubber is mixed directly with hot bitumen (typically at temperatures between 160 °C and 180 °C) before being combined with aggregates to form asphalt. The rubber is blended into the bitumen to create a homogenous mixture [13,14].

The required performance grade of bitumen for specific climate regions depends on various factors, including temperature ranges, traffic loads, and pavement conditions. Neat bitumen (NB) and CR-TMB are graded based on the Superpave Performance Grading (PG) system [15], which classifies bitumen based on its high- and low-temperature performance characteristics. The required performance grade (PG) of bitumen for specific regions in Malaysia is derived through a combination of climate data analysis and laboratory testing, according to the following procedure:

- Climate analysis: air temperature data for five representative regions of Malaysia are collected (i.e., Kedah, Selangor, Johor, Sabah, and Cameron Highlands), including historical temperature records, and the extreme high and low temperatures that the bitumen will be exposed to are identified on the basis of numerical and regression models, respectively. These temperatures correspond to the limits of low-temperature cracking and high-temperature rutting (design temperatures).
  Laboratory testing: performance tests on NB and CR-TMB samples are conducted to determine their rheological properties at different temperatures and aging stages. The key tests include the dynamic shear rheometer (DSR) test for medium temperature behavior and the rotational viscometer (RV) test for high-temperature behavior. These tests conducted on unaged and short-term-aged samples provide data on parameters such as stiffness, viscosity, and elastic properties. The bending beam rheometer (BBR) test provides a measure of low-temperature stiffness and relaxation properties of asphalt binders. These parameters for long-term-aged samples give an indication of an asphalt binder’s ability to resist low-temperature cracking. For a closer insight into the aging process, attenuated total reflectance-Fourier transform infrared spectroscopy (ATR-FTIR) is applied to both the neat and modified bitumen.
- Superpave performance grading (PG): the results from the laboratory testing are used to assign the appropriate performance grade based on the Superpave PG system. The PG grading system categorizes bitumen based on its stiffness and resistance to temperature-induced distress.

Based on the results of the rheological and spectroscopic bitumen characteristics and the derived PGs for the NB and CR-TMB, the improved performance as well as the expected engineering behavior of the CR-TM modification are discussed, taking into account the relevant design temperatures obtained for Malaysian climate regions.

## 3. Design Temperature for Malaysian Regions

### 3.1. Derivation of Critical Pavement Temperature from Air Temperature Data

Design pavement temperatures for Malaysian regions are calculated from air temperature data. Environmental conditions are specified according to the SHARP Superpave grading system in terms of (i) average 7-day maximum pavement design temperature and (ii) minimum pavement design temperature. The average 7-day maximum pavement design temperature is the average of the highest daily pavement temperatures for the 7 hottest consecutive days in a year. The lowest annual pavement temperature is the coldest temperature of the year. The process of calculating pavement temperature from air temperature is as follows: (i) define the boundary conditions at the pavement surface given by the theoretical energy balance according to equation (2) and (ii) calculate temperature profiles using the finite difference method (FDM), (iii) extract 7-day maximum pavement temperature at design depth, and (iv) extract minimum pavement temperature at design depth.

The boundary conditions of the theoretical energy balance equation used to calculate the pavement temperature distribution from air temperature are as follows:

(i) surface temperature: the surface of the pavement is subjected to the convective heat flux from the air, solar radiation, and longwave radiation exchanges. At the surface, the pavement temperature is calculated based on these energy inputs, with the assumption that the heat flux into the pavement is equal to the heat flux out of it.
(ii) subsurface temperature: at the bottom of the pavement structure, a constant subsurface temperature is assumed, typically based on the annual average ground temperature at that depth. For Malaysia regions, a constant temperature of 25 °C in a depth of 5.0 m is assumed. This boundary condition reflects the thermal stability of the deeper subgrade soil layer, where the effect of surface temperature fluctuations diminishes with depth.

**Figure 1:**
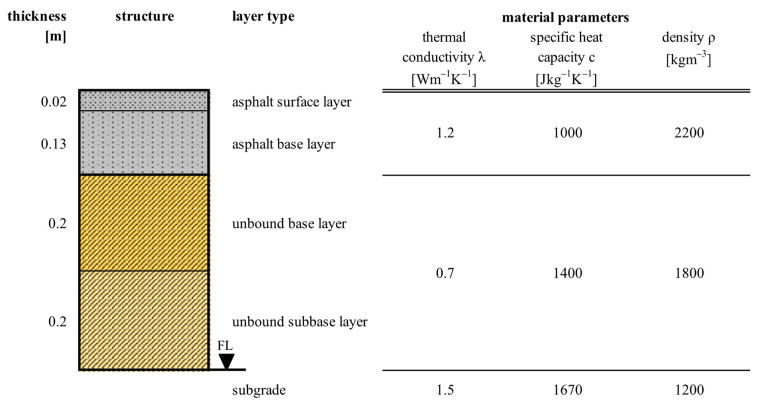
Representative Malaysian pavement structure and material parameters of road layers for thermal diffusivity study.

The design depth for calculation of maximum pavement temperature used in the Superpave system is 20 mm below the top of the respective asphalt pavement layer. Considering a typical Malaysian pavement structure shown in Figure 1, the design depth is 20 mm (temperature @ 0.02 m: T0.02) for the asphalt surface layer ACsurf and 60 mm (temperature @ 0.06 m: T0.06) for the asphalt base layer ACbase, respectively.

To determine the pavement surface temperature from the air temperature and the corresponding temperature distribution within the pavement structure, a numerical model is employed to calculate the design temperature. This model was validated across various road test sites and climate conditions using hourly air temperature and pavement temperature measurements at different layers and depths to ensure accuracy (details are provided in [16]). The thermal parameters of road materials used in the model were set according to [16] for typical pavement structures and tropical soils and are given in Figure 1.

The diffusion equation,

(1) $$\frac{\partial T}{\partial t}=a\;\frac{\partial^{2}T}{\partial x^{2}}$$

a first-order homogeneous differential equation with constant coefficients, is solved using the finite difference method (FDM) with an implicit scheme [17]. As suggested in the Superpave Performance Grading (PG) system, a theoretical energy balance is defined at the pavement surface with:

(2) $$Q-\lambda \frac{\partial T}{\partial x}-h_{c}\left(T_{A}-T_{S}\right)=0$$

where Q represents the radiation balance in Wm^−2^, λ the thermal conductivity Wm^−1^ K^−1^, x the spatial coordinate in pavement depth, h_c_ the surface coefficient of heat transfer, T_s_ the surface temperature in K, and T_a_ the air temperature in K. The radiation balance Q at the pavement surface is the sum of shortwave radiation and longwave radiation and is defined by:

(3) $$Q=Q_{sw}+Q_{lw}$$

**Figure 2:**
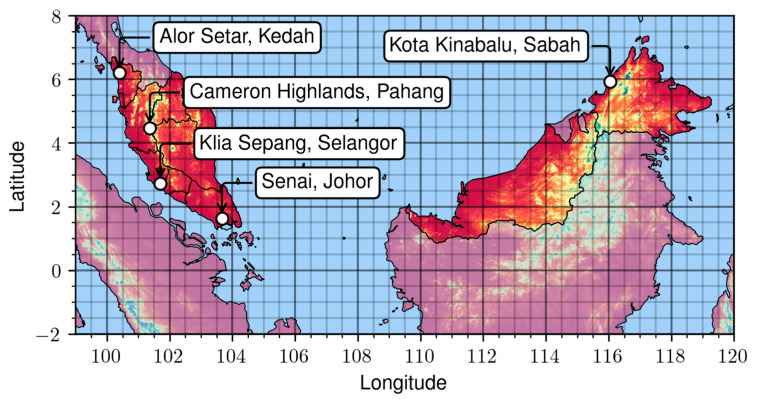
Weather stations representative for Malaysian climate regions.

Shortwave radiation is the incoming solar radiation that reaches the pavement surface and is influenced by the albedo of the surface and the solar zenith angle, and it is estimated by [18]

(4) $$Q_{sw}=\alpha \cdot \left(910\cos z-30\right)$$

where α represents the albedo of the asphalt and z the solar zenith angle. The longwave radiation is defined by:

(5) $$Q_{lw}=\varepsilon_{a}\sigma T_{a}^{4}-\varepsilon \sigma T_{s}^{4}$$

where ε_a_ represents the emissivity coefficient of the air, σ the Stefan–Boltzmann constant, T_a_ the air temperature in K, ε the emissivity coefficient of the pavement surface, and T_s_ the surface temperature in K. The hourly air temperature data from 5 representative weather stations in Malaysia, as shown in Figure 2, are used as input data in this model.

**Figure 3:**
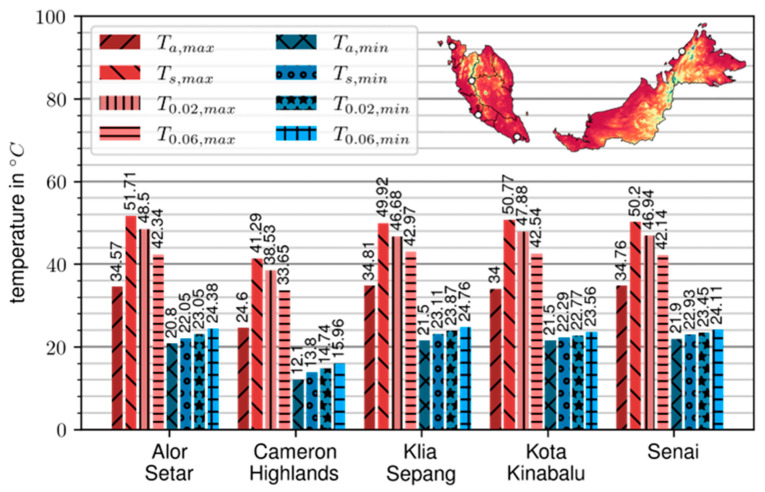
Design temperatures of representative for Malaysian climate regions.

The results of the numerical simulation for pavement surface and design temperatures are summarized in Figure 3.

Under the given assumptions, surface temperatures in four representative regions of Malaysia—Kedah, Selangor, Johor, and Sabah—vary between 22 °C (T_s,min_) and 52 °C (T_s,max_) throughout the year. However, in the Cameron Highlands (Pahang region), the minimum design surface temperature drops to 14 °C (T_s,min_), while the maximum temperature only reaches 42 °C (T_s,max_).

### 3.2. PG Binder Grade for Malaysian Regions

The high pavement design temperature was defined in the SUPERPAVE PG system as the mean seven-day maximum pavement temperature determined at a depth of 20 mm below the pavement surface, a depth where the combined effects of temperature and load-induced shear stress were critical [19]. Low pavement design temperature was defined as the minimum pavement temperature observed in a year at the pavement surface. PG binder grades occur in six-degree increments.

The Superpave PG system was originally calibrated for highway traffic with an average speed of approximately 100 km/h and a maximum load of 10 million equivalent single axle loads (MESALs) [19]. However, certain locations—such as intersections, uphill sections, toll booths, and bus stops—experience slower loading rates or even stationary loading. In these areas, the binder must be stiffer to minimize rutting. Therefore, under severe traffic conditions, it is necessary to increase the high PG binder grade, a process commonly referred to as grade “bumping”. No adjustment is required for the low PG binder grade.

Furthermore, the SUPERPAVE models incorporate reliability into the selection of PG binder grades. This reliability reflects the probability that, in any given year, the actual one-day low air temperature or seven-day mean high air temperature will not exceed the corresponding pavement design temperatures. Agencies determine the appropriate level of reliability based on factors such as road classification and traffic volume. A minimum reliability of 90% is typically used for low-volume rural and residential roads, while a minimum of 95% is standard for high-volume road infrastructure.

The binder performance grade derived from the simulations of the maximum pavement temperatures and their standard deviation for different Malaysian regions is summarized in Table 1.

## 4. Materials and Testing Methods

### 4.1. Neat Bitumen

The neat bitumen used as base bitumen for modification using crumb rubber is of penetration grade 60-70. This bitumen is semi-hard penetration grade bitumen used as paving grade suitable for road construction and repair. In Malaysia, the public works department specifies PEN 60-70 as the most used bitumen grades for the manufacturing of hot mix asphalt for wearing courses and bases. The technical specification of PEN 60-70 bitumen is presented in Table 2 below.

### 4.2. Crumb Rubber Tire Modified Bitumen (CR-TMB)

The crumb rubber tire modified bitumen (CR-TMB) is derived from reclaimed tire rubber, specifically obtained from commercial vehicle tires with an average outer diameter ranging from 660 mm to 1520 mm, commonly seen on trucks and buses. The crumb rubber is characterized and quantified by the mesh screen or sieve size it goes through during the production process. According to the ASTM D8 standard [27], asphalt-rubber is defined as a composite material comprising bitumen, reclaimed tire rubber, and specific additives. In this composition, the rubber component must constitute 15% of the total mixture by weight and undergo a suitable reaction with the heated bitumen, resulting in the expansion of rubber particles.

The CR-TMB is prepared and subjected to increased temperatures and vigorous agitation in order to facilitate the physical interaction between the bituminous binder and the components of reclaimed tire rubber, as well as to ensure the continuous suspension of rubber particles inside the composite material. The CR-TMB contains visible particles of the reclaimed tire rubber. The crumb rubber is obtained by initially cutting it into sizable pieces, which are subsequently shredded into chips measuring around 3 to 5 mm in diameter. The chips undergo initial processing in a coarse mill, followed by further refinement in grinding mills and screens to achieve a mesh size ranging from 20 to 40 (0.8 mm to 0.4 mm).

Table 3 presents the chemical composition of crumb rubber obtained from various tire types or components. The composition of crumb rubber, including parameters such as acetone extract, rubber hydrocarbon content, and natural rubber content, is specified according to ASTM D297 [28]. It is imperative that upon the selection and evaluation of the crumb rubber, the chemical composition remains unchanged.

The crumb rubber tire-modified binders (CR-TMB) are produced by the pre-blending process involving a wet mixing process between the neat binder of penetration grade 60-70, and crumb rubber. This blending process takes place at high temperatures and high shear to promote desulfurization and depolymerization of crumb rubber in the neat bitumen significantly to improve bitumen elasticity [29]. The rubber particles undergo a swelling phenomenon and experience a softening effect as they chemically react with the neat bitumen. The outcome of this reaction is impacted by several factors, including the temperature at which the blending occurs, the duration of high temperature, the method and intensity of mechanical mixing, the characteristics of the crumb rubber, and the aromatic properties of the bitumen.

The process begins by heating the neat bitumen to 170 °C and adding 15% of finely ground crumb rubber particles with a mesh size of less than 40. The crumb rubber is then blended with the neat bitumen at 4000 rpm for 60 min at 190 °C. The rubberized bitumen is then placed in the oven for 30 min at 170 °C for further swelling.

In order to verify the completion of the blending process, a minute quantity of CR-TMB is applied onto a non-permeable surface, revealing a few specks of rubber particles that are soft enough to be manually spread into the CR-TMB using a knife or spatula.

### 4.3. Laboratory Aging

Laboratory short- and long-term aging was conducted to determine the aging behavior of the neat binders and the CR-TMB. For short-term aging, the rolling thin film oven test (RTFOT) was carried out following the ASTM D2872 [30] with the aging temperature of 163 °C and an aging duration of 75 min. For the simulation of the long-term aging, the pressure aging vessel (PAV) test was performed according to the ASTM D6521 [31]. The short-term-aged binder was transferred to the suitable PAV metal containers and oxidized at a temperature of 100 °C and a pressure of 2.1 MPa for a duration of 20 h. The short-term and long-term-aged binders were then used for chemical and mechanical characterization.

### 4.4. Physical Properties of PEN 60/70 and CR-TMB

The penetration and softening point are indicative of the characteristic physical properties of bitumen. The findings for the neat bitumen (NB) and CR-TMB are presented in Table 4. It is evident that CR-TMB exhibits lower penetration values and a higher softening point in comparison to neat bitumen. The interaction between crumb rubber and bitumen results in the absorption of aromatic oil by the crumb rubber particles. This absorption subsequently leads to an increase in the resin and bitumen constituents within the bitumen, as well as an increase in the volume of crumb rubber. The modification of bitumen has led to significant enhancement in its high-temperature properties [32].

### 4.5. Performance Tests

Viscosity is a critical parameter for asphalt materials, playing a vital role in their workability during construction and placement. It represents a material’s resistance to flow and ease of handling, compacting, and shaping during the paving process of asphalt mixtures. In this study, the rotational viscosimeter (RV) is used to measure the dynamic viscosity of NB and CR-TMB (ASTM D4402 [33]). Three readings were taken at temperatures of 120 °C, 135 °C, 150 °C, 165 °C, and 180 °C, and the mean value was reported.

The bending beam rheometer (BBR) is used to assess the performance of asphalt paving binders at low temperatures. The test measures the stiffness (S-value) and relaxation properties (m-value) of bitumen by applying a load to a small beam of the material at low temperatures. The creep stiffness (S) @60s and creep rate (m) @60s are two main outputs of the BBR test (ASTM D6648 [34]). These parameters provide an indication of the bitumen’s capacity to resist cracking at low temperatures.

The S-value indicates the bitumen’s resistance to deformation under load. If the S-value is too high, the bitumen may be prone to cracking under low temperatures because it is too rigid. The m-value indicates the bitumen’s ability to relax stress over time. A high m-value is desirable because it means the bitumen can accommodate thermal stresses without cracking [35].

In this study, the effects of RTFOT and PAV-aged NB and CR-TMB are evaluated by determining the flexural creep stiffness at three temperatures: −12 °C, −18 °C, and −24 °C and −18 °C, −24 °C, and −30 °C, respectively.

The dynamic shear rheometer (DSR) is used to characterize the viscous and elastic behavior of bitumen by means of the specimen’s complex shear modulus (G*) and phase angle (δ) at medium temperatures (ASTM D7552) [36]. The complex shear modulus (G*) characterizes a material’s resistance to deformation when subjected to repeated shearing, expressed as the ratio of peak stress to peak strain. On the other hand, the phase angle (δ) signifies the phase difference between the applied stress and the resulting strain, providing insights into the relative distribution of elastic and viscous responses during bitumen loading [37].

In order to resist rutting, the complex shear modulus elastic portion, G*/sinδ at elevated temperatures should be large. According to [37], when the value of G*/sin δ is larger, the behavior of bitumen resembles that of an elastic material, which is beneficial for enhancing resistance against rutting.

In this study, DSR tests are conducted on unaged and RTFOT-aged NB and CR-TMB samples at seven temperatures, ranging from 40 °C to 82 °C in 6 °C increments.

### 4.6. Chemical Properties via Fourier Transform Infrared (FTIR) Spectroscopy

FTIR spectroscopy is a powerful tool to characterize bitumen in regards to its chemical composition and changes due to modification or aging via infrared active functional groups. Among these groups are carbonyls at 1700 cm^−1^ and sulfoxides at 1030 cm^−1^ that form upon oxidation, thus being very relevant to detect how much a binder has aged. In this study, both neat and CR-TMB were investigated in all available aging states. For the analysis, an Alpha II spectrometer (Bruker Optics Inc, Ettlingen, Germany), equipped with an ATR diamond crystal and a deuterated triglycine sulphate (DTGS) detector, was used. The spectra were recorded in the wavenumber range of 4000–680 cm^−1^ at a resolution of 4 cm^−1^ and 24 scans. A background scan prior to each measurement was conducted on the empty clean ATRM crystal. Four individual specimens were measured with four repeats to obtain a total of 16 spectra per binder. This ensures sufficient repeatability and reproducibility of the method via simple statistical analysis (mean and standard deviation). For spectral evaluation, the 16 spectra were min–max normalized around the main aliphatic region between 3200–2800 cm^−1^ [38]. The normalized spectra were then subjected to two different integration methods: full baseline integration and tangential integration [39]. While the full baseline integration covers all changes in the respective wavenumber domain, the tangential approach merely covers a specific band or formation of a specific functional group. This leads to the result that the tangential method will not include the increase in the fingerprint intensity caused by an increase in the material’s polarity [40]. Since both methods have been used in the literature [39,41], their respective application will be tested to check which method fits the evaluation of CR-TMB best.

The respective aging indices were generated using the following integration limits:

- Carbonyls (AI_CO_): 1660–1800 cm^−1^
- Sulfoxides (AI_SO_): 1079–984 cm^−1^
- Reference aliphatic band (AI_CH3_): 1525–1350 cm^−1^

The received values were then used to calculate the aging index (AIF_TIR_) according to Equation (6)

(6) $${AI}_{FTIR}=\frac{{AI}_{CO}+{AI}_{SO}}{{AI}_{{CH}_{3}}}$$

## 5. Test Results and Interpretation

### 5.1. Viscosity and Workability

The temperature of 135 °C is commonly selected for assessing the viscosity of asphalt materials due to its practical relevance, allowing the test to simulate conditions under which asphalt mixtures are commonly handled and compacted during construction. The Asphalt Institute [42] recommends using a maximum value of about 0.28 +/− 0.03 Pa·s for compaction temperatures; according to the SHRP Superpave specifications [37], the threshold is ≤3.0 Pa·s.

**Figure 4:**
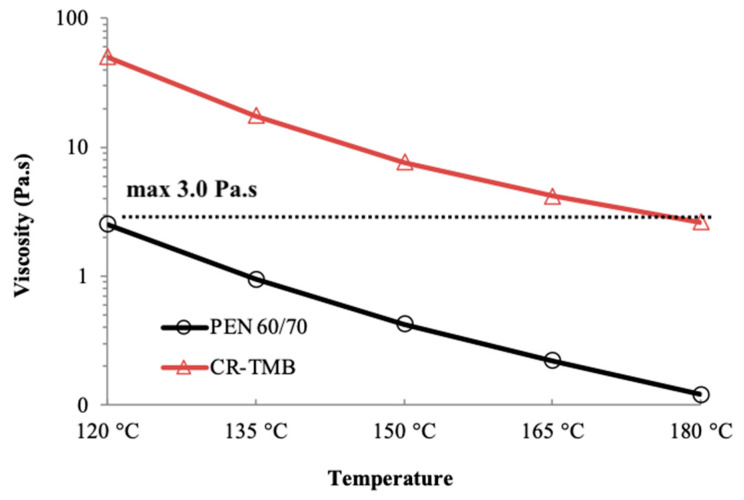
Dynamic viscosity according to ASTM D4402 of PEN 60-70 and CR-TMB.

According to Figure 4, this minimum viscosity value lies under 120 °C for the neat bitumen PEN 60/70 but is met at a significantly higher temperature of 172 °C for the CR-TMB, which indicates a less favorable workability and a small paving window during construction. The detailed data can be found in Table A1 in the Appendix A.

However, the recommended procedures for determining compaction temperatures mentioned above do not apply to bitumen that has been modified with CR-TBM using the traditional “wet process” and which do not meet typical solubility requirements (Asphalt Institute, 2016). Therefore, it is crucial to closely adhere to the supplier’s recommendations and expertise when using this CR-TBM for hot mix asphalt (HMA) at the construction site.

### 5.2. Low Temperature Performance

**Figure 5:**
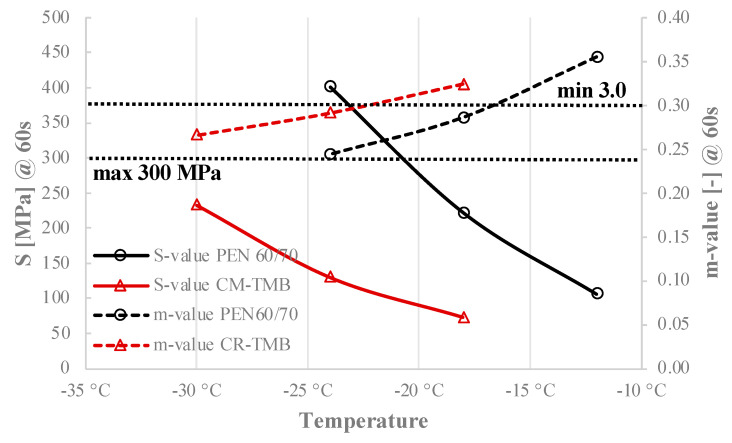
BBR test results (stiffness and m-value) according to ASTM D6648 for RTFOT and PAV-aged PEN 60/70 and CR-TMB.

Figure 5 presents the creep stiffness (S) and creep rate (m-value) obtained from Bending Beam Rheometer (BBR) tests conducted at low temperatures on both the PEN 60/70 and CR-TMB specimens. According to the valid SHRP Specifications [37], the creep stiffness S@60s should possess a magnitude less than 300 MPa, whereas the m-value should have a magnitude more than 0.3. A smaller creep stiffness or a higher m-value would result in better performance at low temperatures. The detailed data can be found in Table A2 in the Appendix A.

The data presented in Figure 5 indicate that the PEN 60/70 exhibits the greatest S value of 401 MPa and the lowest m-value of 0.244 @ −24 °C. Conversely, the CR-TMB has the lowest S value of 73 MPa and a relatively high m-value of 0.324 @ −12 °C. The CR-TMB exhibits a lower m-value compared to PEN 60/70, suggesting that the CR-TMB has a slower rate of stiffness changes under loading conditions in comparison to neat bitumen. The detailed data can be found in Table A3 in the Appendix A.

Test findings demonstrate that the (exact) lower performance grade PG for PEN 60/70 and CR-TMB is −16 °C and −12 °C, respectively. The performance of bitumen under adverse weather conditions demonstrates that the use of CR-TMB can greatly improve its resistance to cracking, particularly in extremely low temperatures.

In the context of tropical regions, this parameter might seem less relevant because these areas typically experience higher ambient temperatures year-round, with only minor fluctuations in temperature. However, understanding and considering the low-temperature performance grade of bitumen in tropical regions can still be important for several reasons: (i) the Malaysian regions are not uniformly hot, as shown in Figure 3; they include areas at higher altitudes where temperatures can drop significantly, especially during the night. In such regions, selecting a bitumen grade with appropriate low-temperature performance can help prevent cracking in the pavement due to thermal contraction, (ii) bitumen with good low-temperature performance generally has higher flexibility; even in tropical climates, where the main concern is typically high-temperature performance (resistance to rutting), ensuring some degree of flexibility helps accommodate stresses from occasional cool nights, heavy loads, or unexpected environmental conditions, and (iii) the longevity of pavements is influenced by the cumulative effects of various stressors, including temperature changes; even small, infrequent drops in temperature can lead to cumulative damage over time if the bitumen is not resilient enough, potentially leading to premature pavement failure.

In conclusion, while the primary concern in tropical regions is indeed high-temperature performance, the low-temperature performance grade of bitumen still plays a role in ensuring overall pavement durability and resilience.

### 5.3. High Temperature Performance

In SHRP Superpave [37], high temperature grade has been defined as the temperature at which G*/sin δ of bitumen before and after RTFOT aging is more than 1.0 and 2.2 kPa, respectively.

**Figure 6:**
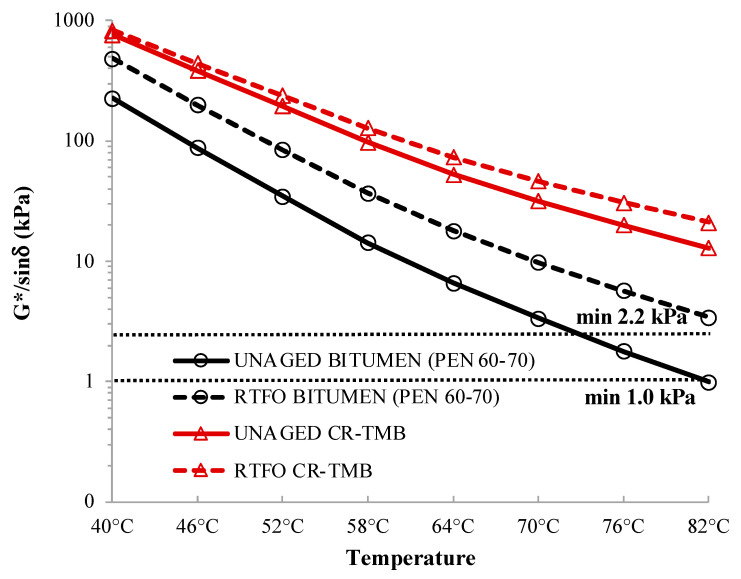
DSR test results (G*/sin δ value) according to ASTM D7552 for unaged and RTFOT-aged PEN 60/70 and CR-TMB.

Figure 6 illustrates the notable enhancement in the G*/sin δ values of CR-TMB resulting from the inclusion of crumb rubber in comparison to neat bitumen. As a result, the conclusion drawn is that the bitumen with crumb rubber exhibits superior rutting resistance and significantly reduces the rutting failure in asphalt pavements when compared to the PEN 60/70. This result has been shown previously for road bitumen containing crumb rubber [5,7,8].

This improvement in G*/sin δ value is attributed to the interaction between bitumen and rubber particles during the wet process, leading to the swelling of rubber particles [29]. As a result of this swelling, the bitumen experienced hardening, which ultimately enhanced its stiffness. Observations indicate that G*/sin δ values for all bitumen specimens exceed 1.0 kPa in their unaged state and 2.2 kPa after RTFO aging. This demonstrates that bitumen satisfies the specifications for Superpave at the selected testing temperature.

Furthermore, the comparison of the DSR data reveals that the stiffness of aged specimens subjected to the RTFOT test was greater in comparison to unaged specimens. This observation indicates that the complex shear modulus (G*) exhibited an increase for both neat bitumen and CR-TMB when compared to unaged specimens. This effect is observed as an increase in the complex modulus (G*) and a decrease in the phase angle (δ). However, the effects of aging are much more pronounced in the unmodified (neat) bitumen, which supports the respective findings from the FTIR spectroscopy analysis as described in the following chapter.

The key results in this context are (i) improved elasticity and higher stiffness at elevated temperatures of CR-TMB, which significantly enhances its resistance to rutting; this is crucial in hot climates where pavements are prone to deformation under heavy loads, (ii) the addition of crumb rubber increases the elastic properties of the bitumen, allowing it to better recover its shape after being deformed by traffic loads; this contributes to the longevity of the pavement under high-temperature conditions, and (iii) CR-TMB helps in retaining elasticity longer, even as the bitumen ages, due to the rubber particles’ ability to absorb stresses and strain energy, thus delaying the embrittlement process.

Overall, CR modification helps mitigate the adverse effects of aging on bitumen by improving its oxidative resistance, maintaining elasticity, and reducing susceptibility to cracking and stiffening. This leads to longer-lasting pavements with better performance regarding rutting and aging over time.

### 5.4. Binder Performance Grade

In Table 5, the performance grade (PG), established through the Superpave specifications, for the NB and CR-TMB, is given.

In the results indicate:

- Neat bitumen (60/70): PG 64-16:
  The neat bitumen with a penetration grade of 60/70 has a PG of 64-16, indicating it can handle temperatures up to 64 °C and down to −16 °C. For Malaysia’s climate and typical traffic conditions, this PG grade is generally sufficient, as it aligns with the standard temperature and load-bearing requirements for the region. This binder would perform well under typical traffic volumes and standard speeds, providing adequate rutting resistance and temperature stability.
- Crumb rubber tire modified bitumen (CR-TMB): PG 82-22:
  The crumb rubber-modified bitumen has a higher PG of 82-22, meaning it can withstand temperatures up to 82 °C and down to −22 °C. This higher performance grade makes it suitable for conditions where there is a need for enhanced resistance to high temperatures and heavy loads, such as in cases of slow-moving or high-volume traffic. The improved high-temperature grade (82 °C) indicates superior resistance to rutting under heavy or prolonged loading, which would help in preventing deformation in high-stress conditions.

In conclusion, the NB PG 64-16 is adequate for standard traffic conditions; CR-TMB with PG 82-22 would be necessary for higher traffic volumes (over 10 million equivalent single-axle loads, MESALs) or areas with slow-moving traffic, where the binder is subject to increased stress and temperature. The CR-modified bitumen provides better high-temperature performance, reducing the risk of rutting and prolonging pavement lifespan under such demanding conditions. Therefore, CR-TMB presents a viable solution for large-scale road construction projects, particularly in high-traffic and high-temperature environments.

### 5.5. Chemical Composition (Fourier Transform Infrared) (FTIR) Spectroscopy

**Figure 7:**
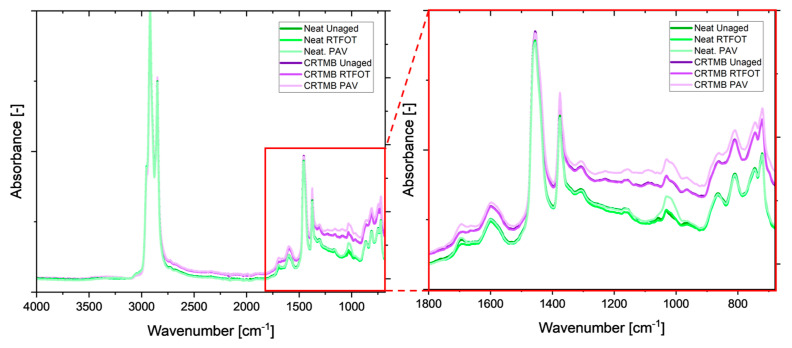
FTIR spectra of all neat and CR-TMB at full range (**left**) and fingerprint region (**right**).

Figure 7 shows the FTIR spectra of all NB and CR-TMB in the unaged, short-term, and long-term-aged states. By comparing the green (neat) and purple (CR-TMB) spectra on the right side of Figure 7, one can see the influence of the CR modification. Besides the overall increase in the fingerprint region, shifting to higher absorbance, no obvious additional bands can be detected. This means that mostly the bitumen-specific bands associated with alkyl groups at 2920, 2850, 1460, 1380, and 720 cm^−1^, as well as the bands assigned to aromatic structures at 1600, 860, 810, and 750 cm^−1^ can be seen beside the two functional groups (carbonyls at 1700 and sulphoxides at 1030 cm^−1^).

Similar results are reported in the literature, where work by Xiang and Nivitha shows no additional infrared (IR) active functional group after the addition of the crumb rubber [43,44]. The significant increase in the fingerprint intensity was typically linked to a shift towards higher polarity upon aging [40]. However, this shift can solely be explained by the addition of the crumb rubber and can thus not be explained based on FTIR spectroscopy. To investigate this phenomenon and confirm this hypothesis, further analysis would be needed.

**Figure 8:**
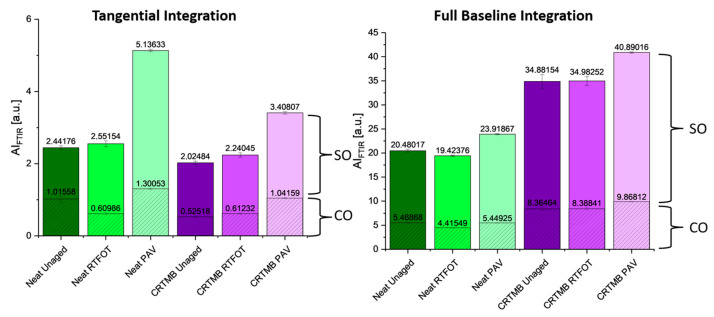
FTIR aging indices from the tangential integration method (**left**) and the full baseline integration method (**right**).

The evaluation of the FTIR aging indices is shown in Figure 8. The difference between the two integration methods becomes obvious when considering the difference in the fingerprint intensity, which is caused by the addition of the CR. When considering the full baseline integration, it can be assumed that the aging level of the CR-TMB binder is higher. However, this might be a false assumption, as this intensity increase is solely caused by the addition of the CR. Thus, the tangential integration method might be more suitable to evaluate the actual formation of carbonyls and sulfoxides (1030 and 1710 cm^−1^, respectively; see the above reference). The results show that the CR-TMB incorporates fewer oxidized functional groups compared to the neat binder. However, as mentioned earlier, this hypothesis would need confirmation via additional analysis.

A possible explanation could be given by the fact that physical CR particles are included into the binder, which might retard the diffusion of oxygen into the material, thus slowing down the oxidative aging process in bitumen. This leads to a slower rate of hardening and reduced brittleness over time, which helps maintain the flexibility and durability of the pavement. The enhanced aging resistance provided by CR-TMB makes bitumen more resilient to environmental factors like UV radiation, ozone, and other air pollutants, which are significant contributors to the aging process. The rubber particles shield the bitumen from some of these effects, further prolonging its service life.

## 6. Conclusions

This research aimed to determine the appropriate performance grade of neat bitumen (NB) with a penetration grade of 60/70, as well as crumb rubber-modified bitumen (CR-TMB) produced using the wet process, in accordance with the Superpave PG system under Malaysian climate conditions. The study was conducted on a restricted number of bitumen samples, which may impact the generalizability of the findings, and based on the design, pavement temperatures are specific to the Malaysian and comparable tropical regions. The findings led to the following conclusions:

1. Design Temperatures
  Malaysia, being a tropical country, experiences high temperatures and heavy rainfall. Conventional asphalt can become soft and rut under high temperatures, and the water can cause stripping. The design temperatures play a crucial role in determining the performance grade of the binder used.
  Therefore, pavement design temperatures for five representative Malaysian regions were determined using hourly air temperature data. This was done by simulating the temperature distribution within a typical Malaysian road structure, employing the finite difference method (FDM) with an implicit scheme. As a result, the appropriate design temperatures (PGs) can be recommended for different regions of Malaysia and varying traffic volumes.
2. Binder Rheology
  CR-TMB has been shown to have lower thermal susceptibility compared to PEN 60/70, along with significantly higher viscosity at elevated temperatures. This means that while workability and compaction at the construction site are more sensitive, roads built with CR-TMB are less likely to soften excessively during hot days or become brittle at night. CR-TMB offers a broader range of temperatures over which it can perform optimally. This means that the roads can maintain their structural integrity and performance over a wider temperature range.
  One of the primary benefits of using CR-TMB is its enhanced rut resistance. Given Malaysia’s high temperatures, rutting can be a significant concern. CR-TMB, with its modified properties, can provide better resistance against deformation under heavy traffic loads. CR-TMB demonstrates enhanced resilience to temperature variations, showing significant improvements in both high- and low-temperature conditions.
3. Binder Aging and Performance
  The inclusion of crumb rubber in bitumen (CR-TMB) significantly influences its aging behavior and performance. In terms of aging behavior, Fourier transform infrared (FTIR) spectroscopy indicates that the crumb rubber’s addition doesn’t introduce new functional groups but alters the fingerprint intensity. This change is predominantly due to the rubber addition rather than the aging process.
  As shown in previous studies [29], the modified binder properties in CR-TMB can improve adhesion between the aggregate and the binder. This improved adhesion can reduce the chances of water-induced damage, which is crucial given Malaysia’s heavy rainfall patterns. However, moisture sensitivity was not specifically investigated in this study. Together with the improved resistance against rutting, temperature susceptibility, and water damage, roads constructed using CR-TMB are expected to have a longer service life. This can lead to reduced maintenance costs in the long run.
4. Environmental Benefits
  Using crumb rubber in the binder utilizes waste tires that otherwise pose an environmental disposal challenge. By using recycled rubber from scrap tires, CR-TMB addresses environmental concerns, reducing waste and the demand for virgin materials. This sustainable approach can be a significant benefit in regions seeking greener construction practices. This not only provides a sustainable solution for waste tire management but also reduces the carbon footprint of road construction. The processing of crumb rubber into bitumen generally requires less energy compared to producing new materials, leading to lower greenhouse gas emissions during manufacturing. Due to improvements in rheology and aging behavior, roads made with CR-TMB are more durable, wear resistant, and require less frequent maintenance. This extended lifespan reduces the need for resource-intensive repairs and reconstruction, thereby lowering the environmental impact of road maintenance activities.
5. Potential Limitations and Considerations
  While CR-TMB offers multiple performance benefits, its behavior under extreme traffic and climate conditions warrants further research. Additionally, implementing CR-TMB in large-scale projects requires consideration of regional factors, such as local availability of materials and compatibility with existing construction practices. The success of CR-TMB also depends on optimal design and application practices, as the composition and distribution of crumb rubber can impact performance.

In conclusion, considering Malaysia’s climatic conditions and the challenges posed by high temperatures and heavy rainfall, the use of CR-TMB in asphalt road constructions offers promising improvements in terms of performance, durability, and sustainability. Further research is essential to explore the performance of CR-TMB asphalt mixture with respect to variations in traffic load and moisture-induced damage on the pavement. Additionally, a broader number of bitumen samples would be desirable to strengthen the findings and enhance the reliability of the results. The focus should be on identifying the most effective formulations and methods to enhance durability and performance under varied tropical conditions, as well as on practical implementation in road projects.

## Figures and Tables

**Table 1:** Binder performance grade required for Malaysian regions.

Malaysian Region	Binder Performance Grade ^(a)^	
	90% Reliability	95% Reliability
Alor Seta, Klia SepangKota Kinabalu, Senai	PG 52-10	PG 58-10
Cameron Highlands	PG 46-10	PG 52-10

^(a)^ Increase upper grade 6 °C for > 3 MESALs or slow traffic and 12 °C for >10 MESALs.

**Table 2:** Neat bitumen (PEN 60/70) technical specification.

Bitumen 60/70	Test Method	Unit	Specification
Specific gravity @ 25 °C	ASTM D70 [20]	g/cm^3^	1.01–1.06
Penetration @ 25 °C	ASTM D5 [21]	mm/10	60–70
Softening Point	ASTM D36 [22]	°C	49–56
Ductility @ 25 °C	ASTM D113 [23]	cm	100 min
Loss on heating	ASTM D6 [24]	wt%	0.2 max
Flashpoint	ASTM D92 [25]	°C	232 min
Solubility in trichloroethylene	ASTM D2042 [26]	wt%	99 min

**Table 3:** Chemical composition of crumb rubber.

Test Parameter	Specification
Acetone extract (%)	<22
Rubber hydrocarbon (%)	42–65
Carbon black content (%)	28–38
Natural rubber content (%)	>30
Ash content (%)	<8

**Table 4:** Physical properties of Pen 60-70 and CR-TMB.

Properties	Unit	Neat Bitumen (PEN60/70)		CR-TMB	
		Unaged	RTFO Aged	Unaged	RTFO Aged
Penetration @25 °C	0.1 mm	61.1	41	33	25
Softening point	°C	49.4	55.4	76.6	87

**Table 5:** Performance grade of neat and CR modified bitumen.

Binder	Performance Grade
60/70	PG 64-16
CR-TMB	PG 82-22

## Data Availability

The original contributions presented in the study are included in the article, further inquiries can be directed to the corresponding author.

## Appendix A. Data Tables

**Table A1:** Viscosity tests at different temperatures.

Viscosity (mPas)		Temperature (°C)	Average Viscosity (mPas)		Average Temperature (°C)
Neat Bitumen (PEN60-70)	CRMB		Control	CRMB	
2525	51,250	120	2521	50,111	120
2525	49,167	120			
2512.5	49,917	120			
950	17,075	135	946	17,525	135
950	18,450	135			
937.5	17,050	135			
425	7413	150	425	7613	150
437.5	7675	150			
412.5	7750	150			
225	4225	165	221	4167	165
225	4088	165			
212.5	4188	165			
125	2600	180	121	2621	180
125	2625	180			
112.5	2638	180			

**Table A2:** Flexural creep stiffness and creep rate of bitumen.

Bitumen Type	Temperature (°C)	Flexural Creep Stiffness (S) (MPa) S < 300 MPa	m-Value [-] @ 60 s m > 0.3
NEAT BITUMEN	−12	106	0.335
	−18	222	0.286
	−24	401	0.244
CR-TMB BITUMEN	−18	73	0.324
	−24	130	0.291
	−30	233	0.266

**Table A3:** Complex modulus (G*) and phase angle (δ) values for both unaged and RTFO-aged bitumen in both neat bitumen and CR-TMB mixes.

Bitumen	Test Temperature (°C)	Complex Modulus G* (KPa)	Phase Angle δ (°)	Rutting Parameter
				G*/sin δ (kPa)
NEAT BITUMEN (PEN 60/70)	40	205.72	68.1	227.4
	46	82.04	72.2	87.8
	52	33.06	76.0	34.7
	58	13.81	80.6	14.2
	64	6.48	82.8	6.6
	70	3.31	84.3	3.3
	76	1.77	85.1	1.8
	82	0.98	85.4	1.0
CR-TMB	40	407.30	60.7	482.0
	46	174.41	63.6	198.3
	52	76.10	63.3	84.5
	58	33.62	65.4	36.8
	64	16.57	68.0	17.9
	70	9.03	68.7	9.7
	76	5.27	68.5	5.7
	82	3.15	69.0	3.4
UNAGED CR-TMB	40	550.72	45.9	768.7
	46	285.58	47.4	385.8
	52	147.74	48.7	195.1
	58	75.25	50.0	97.4
	64	41.00	51.2	52.4
	70	24.91	52.4	31.5
	76	15.89	53.4	19.9
	82	10.42	54.2	12.9
RTFO CR-TMB	40	559.44	42.1	828.6
	46	305.31	43.1	440.8
	52	167.56	43.7	238.2
	58	91.53	44.6	128.1
	64	52.61	45.3	73.2
	70	33.60	46.1	46.4
	76	22.31	46.8	30.6
	82	15.41	47.6	20.9
